# Crystal structure of a mononuclear copper(II) complex with 2-methoxy-*N*,*N*-bis­(quinolin-2-yl­meth­yl)­ethyl­amine (DQMEA)

**DOI:** 10.1107/S2056989018010319

**Published:** 2018-07-24

**Authors:** Steven T. Frey, Jason Li, Manpreet Kaur, Jerry P. Jasinski

**Affiliations:** a Department of Chemistry, Skidmore College, 815 North Broadway, Saratoga Springs, NY 12866, USA; b Department of Chemistry, Keene State College, 229 Main Street, Keene, NH 03435-2001, USA

**Keywords:** crystal structure, copper(II), tripodal ligand, Jahn–Teller distortion

## Abstract

Structural characterization of the compound [Cu(C_2_H_3_N)(C_23_H_23_N_3_O)](ClO_4_)_2_] or [Cu(C_2_H_3_N)(DQMEA)](ClO_4_)_2_] [DQMEA = 2-methoxy-*N*,*N*-bis­(quinolin-2-ylmeth­yl)ethyl­amine] {systematic name: (aceto­nitrile)[2-methoxy-*N*,*N*-bis(quinolin-2-ylmeth­yl)ethyl­amine]­copper(II) diperchlorate} by single-crystal X-ray diffraction reveals a complex cation with a tetra­dentate coordination of the DQMEA ligand along with monodentate coordination of a CH_3_CN ligand to a single Cu^II^ center, with two perchlorate anions providing charge balance. The Cu^II^ center has a distorted square-pyramidal geometry in which the nitro­gen atoms of the DQMEA and CH_3_CN ligands occupy the equatorial positions, while the oxygen atom of the DQMEA ligand resides in the axial position with an elongated Cu—O bond. The quinoline ring systems are nearly co-planar in the structure, while the linear CH_3_CN ligand is tilted significantly below this plane, and the central nitro­gen of DQMEA is above it. Within the complex, weak C—H⋯N hydrogen bonding takes place between the nitro­gen of CH_3_CN and a neighboring quinolyl group. The perchlorate ions are disordered within the structure, but undergo a number of weak inter­molecular C—H⋯O hydrogen-bonding inter­actions. Additional weak π-stacking inter­actions between the quinolyl groups of neighboring complexes further stabilize the crystal packing.

## Abstract 2

The crystal structure of [Cu(DQMEA)(CH_3_CN)](ClO_4_)_2_ (DQMEA = *N*,*N*-bis­(2-quinolylmeth­yl)meth­oxy­ethyl­amine) has been determined. The structure reveals a five-coordinate Cu^II^ center with a distorted square-pyramidal geometry.

## Chemical context   

Copper proteins are numerous in living systems, owing largely to their ability to bind and process di­oxy­gen (Karlin & Tyeklár, 1993 ▸; Karlin, 1993 ▸; Kopf & Karlin, 1999 ▸). Much of what is known about these proteins comes from modeling studies that involve the synthesis of low mol­ecular weight copper complexes with organic-based ligands (Mirica *et al.*, 2004 ▸; Lewis & Tolman, 2004 ▸; Hatcher & Karlin, 2004 ▸; Peterson *et al.*, 2013 ▸). Many of these involve N-centered, tripodal, tetra­dentate ligands containing pyridine or quinoline moieties (Wei *et al.*, 1994 ▸; Young *et al.*, 1995 ▸; Kim *et al.*, 2015 ▸). These ligands give stable complexes that provide access to both the Cu^I^ and Cu^II^ oxidation states, and leave open or solvent-bound coordination sites for the binding of di­oxy­gen species (Wei *et al.*, 1994 ▸).

More recently, copper(II) complexes have been targeted as potential anti­cancer agents (Santini *et al.*, 2014 ▸). Indeed, copper(II) has been shown to promote tumor cell death through a variety of mechanisms while remaining less toxic systematically than platinum-based drugs (Angel *et al.*, 2017 ▸). A number of the compounds that have been studied employ pyridyl, quinolyl, and other aromatic amine-containing ligands because of their ability to form stable complexes with copper(II) ions that display promising anti­cancer activity (Angel *et al.*, 2017 ▸; Santini *et al.*, 2014 ▸). Given the rich variety of ligands of this type, copper(II) complexes with a range of coordination numbers, geometries, redox potentials, biological compatibility, and cytotoxicity are possible.
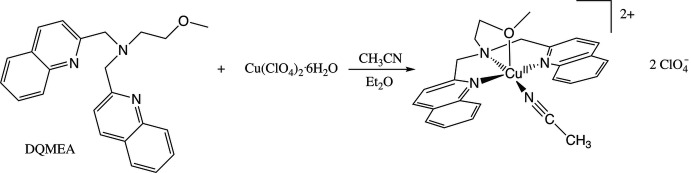


Based on their relevance to biology, we have begun to explore copper(II) complexes with novel *N*-tripodal ligands containing either pyridine or quinoline moieties. We report here the synthesis and structural characterization of [Cu(DQMEA)(CH_3_CN)](ClO_4_)_2_] [DQMEA = 2-methoxy-*N*,*N*-bis­(quinolin-2-ylmeth­yl)­ethyl­amine]. This compound is formed by the reaction of copper(II) perchlorate with DQMEA in aceto­nitrile, followed by the addition of diethyl ether (see reaction scheme) to afford dark-blue crystals suitable for X-ray diffraction studies.

## Structural commentary   

**Figure 1:**
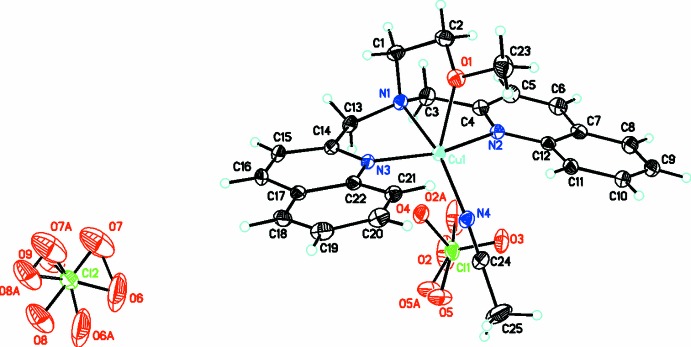
Structure of the title compound, [Cu(DQMEA)(CH_3_CN)](ClO_4_)_2_, with atom labels, shown with displacement ellipsoids drawn at the 30% probability level. Both perclorate anions are disordered, with oxygen occupancy ratios of 0.900 (10):0 l.100 (10) and 0.779 (16):0.319 (7).

The title compound (Fig. 1 ▸) crystallizes in the monoclinic *P*2_1_/*n* space group. The structure reveals a monomeric cation of [Cu(DQMEA)(CH_3_CN)]^2+^ with two disordered perchlorate counter-anions. The copper(II) center is penta­coordinate with a distorted square-pyramidal geometry as indicated by the trigonality index, τ = 0.03 defined as τ = |θ − φ|/60, where θ and φ are the two largest angles in the coordination sphere (Addison *et al.*, 1984 ▸). These angles are 164.97 (8) and 163.04 (9)° (Table 1 ▸). According to this index, τ values of five-coordinate complexes range from 0 for perfectly square-planar to 1 for perfectly trigonal–bipyramidal geometries. The DQMEA ligand is tetra­dentate with its central (N1) nitro­gen and two quinolyl nitro­gen atoms (N2 and N3) lying in the equatorial plane, and the meth­oxy oxygen atom (O1) taking up the axial position. The fourth position in the equatorial plane is occupied by the nitro­gen atom (N4) of a coordinated aceto­nitrile mol­ecule. The two quinoline ring systems of DQMEA are nearly co-planar with each other [dihedral angle = 14.58 (7)°], which results in a steric inter­action between hydrogen atoms H11 and H21 and the coordinated aceto­nitrile mol­ecule. This causes the linear aceto­nitrile mol­ecule to drop below the quinolyl plane, such that the bond angle that its nitro­gen atom makes with the copper ion and the axial oxygen of DQMEA, O1—Cu1—N4, is 115.14 (8)°. The bite angles imposed by the tetra­dentate chelation of the DQMEA ligand cause further constraints leading to some distortion of the structure. For example, the central nitro­gen and meth­oxy oxygen atoms, spanning the equatorial and axial positions, form a five-membered metallocycle with an N1—Cu1—O1 bond angle of 81.40 (8)°. This moves N1 slightly above the quinolyl plane, and causes the non-linearity of N2—Cu1—N3 [164.97 (8)°]. The equatorial bond angles N1—Cu1—N2 [84.06 (8)°] and N1—Cu1—N3 [80.92 (8)°] are also significantly reduced from 90° because of the constraints of the DQMEA coordination. The equatorial Cu—N bond lengths fall in the narrow range of 1.968 (2) to 2.0311 (19) Å, consistent with values reported previously (Wei *et al.*, 1994 ▸), while the axial Cu—O bond is significantly longer at 2.3570 (19) Å. The latter is consistent with a weak axial inter­action due to Jahn–Teller distortion as noted previously for square-pyramidal copper(II) complexes (Chavez *et al.*, 1996 ▸; Warda, 1998 ▸; Rowland *et al.*, 2002 ▸; Roy *et al.*, 2011 ▸). Finally, a weak intra­molecular C—H⋯N hydrogen-bonding inter­action takes place between a quinolyl hydrogen (H11) and the nitro­gen atom of the aceto­nitrile ligand (N4), which may help stabilize the coordin­ation of this monodentate ligand.

## Supra­molecular features   

**Figure 2:**
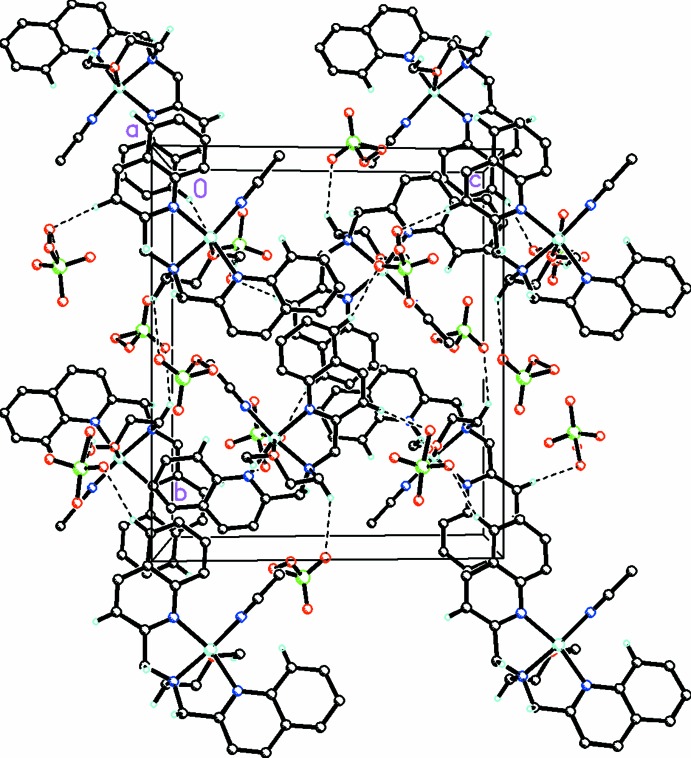
Crystal packing of the title compound viewed along the *a* axis. The inter­molecular C—H⋯O hydrogen bonds (Table 2 ▸) are shown as dashed lines. π–π stacking of the quinoline rings along the *a* axis can be seen in the center of the diagram.

Within the crystal, a network of weak C—H⋯O hydrogen-bonding inter­actions (Table 2 ▸) takes place between the hydrogen atoms of the DQMEA ligand and the oxygen atoms of the perchlorate anions (Fig. 2 ▸). In addition, weak π–π stacking inter­actions between nearby pyridine rings (*Cg*5⋯*Cg*5) of a quinoline group and between the pyridine and phenyl rings (*Cg*5⋯*Cg*7) of other nearby quinoline groups (where *Cg*5 and *Cg*7 are the centroids of the N3/C14–C17/C22 and C17–C22 rings, respectively) serve to further stabilize the crystal packing.

In addition, weak slipped parallel C—H⋯π-ring [C8—H8⋯*Cg*7, *X*—H, π = 50°; C19—H19⋯*Cg*6, *X*—H, π = 47°] and *Y*—*X*⋯*Cg* [Cl1—O3⋯*Cg*4, *X*—H, π = 27.35° and Cl1—O2*A*⋯*Cg*4, *X—*-H, π = 3.33°, where *Cg*4 = N2/C4/C5/C6/C7/C12 and *Cg*6 = C7–C12] inter­molecular inter­actions (Table 2 ▸) are also present and contibute additionally to the crystal packing.

## Database survey   

To the best of our knowledge, a structure of the title compound has not been published previously. However, analogous structures of copper(II) complexes with tripodal ligands formed by tethering two quinolyl groups to either a chiral amino alcohol or amino acid have been reported (Holmes *et al.*, 2005 ▸; Zahn *et al.*, 2006 ▸). Within these chiral structures, the quinolyl groups are not coplanar, but are instead twisted relative to each other in a propeller-like fashion.

## Synthesis and crystallization   

All chemicals were obtained from commercial sources and used without further preparation. Deionized water was used throughout. The ^1^H NMR spectrum was recorded with a JEOL ECX-300 NMR spectrometer and referenced against the ^1^H peak of the chloro­form solvent. IR spectra were recorded with a Perkin Elmer Spectrum 100 FT–IR.

**2-Methoxy-*N*,*N*** **-bis(quinolin-2-ylmeth­yl)­ethyl­amine (DQMEA)**. In a 250 mL round-bottom flask, 5 g (23 mmol) of 2-chloro­methyl­quinoline hydro­chloride was dissolved in 10 mL H_2_O and cooled to 273 K in an ice bath. A solution of 1.9 g (47 mmol) of NaOH in 10 mL H_2_O was added dropwise under stirring. Following this, a solution of 0.9 g (12 mmol) of 2-meth­oxo­ethyl­amine in 10 mL CH_2_Cl_2_ was added. The reaction mixture was then removed from the ice bath, and brought to reflux. After seven days, the mixture was cooled to room temperature and the CH_2_Cl_2_ layer was separated, washed twice with brine, and dried over anhydrous sodium sulfate. The solution was then filtered, and the filtrate was chromatographed on alumina (chromatographic grade, 80–200 mesh) eluting with 20:1 CH_2_Cl_2_/methanol. Fractions were collected that produced a single spot by TLC on alumina plates (eluting with 100:1, CH_2_Cl_2_/methanol) with an *R*_F_ value of 0.33. Rotary evaporation of these fractions gave 2.4 g (58%) of a light-yellow solid. ^1^H NMR (CDCl_3_, 300 MHz) δ 2.87 (*t*, 2H), 3.25 (*s*, 3H), 3.54 (*t*, 2H), 4.09 (*s*, 4H), 7.45 (*t*, 2H), 7.66 (*t*, 2H), 7.75 (*m*, 4H), 8.01 (*d*, 2H), 8.10 (*d*, 2H).

**[Cu(DQMEA)(CH_3_CN)](ClO_4_)_2_]**. In a 50 mL round-bottom flask, 0.100 g (0.28 mmol) of copper(II) perchlorate hexa­hydrate and 0.104 g (0.28 mmol) of DQMEA were dissolved in 10 mL of aceto­nitrile. The reaction mixture was capped and allowed to stir for 30 minutes. Approximately 10 mL of anhydrous diethyl ether was added until crystals began to form on the side of the flask, and the mixture was capped and placed in a refrigerator. After seven days, 0.15 g (84%) of dark-blue crystals suitable for X-ray diffraction were collected by filtration and washed with diethyl ether. IR (ATR, cm^−1^) 2800–3200 (aromatic C—H, *w*), 1604, 1516, and 1436 (aromatic C—C, *m*), 1064 (ClO_4_^−^, *s*, br), 781, 843 (aromatic C—H, *s*).

## Refinement   

Crystal data, data collection and structure refinement details are summarized in Table 3 ▸. All H atoms were positioned geometrically and refined using a riding model: C—H = 0.93–0.97 Å, with *U*_iso_(H) = 1.2*U*_eq_(C) or 1.5*U*_eq_(C-meth­yl). Both perchlorate ions were disordered [occupancy ratios of 0.900 (10):0.100 (10) and 0.656 (7):0.348 (7)].

## Supplementary Material

Crystal structure: contains datablock(s) I. DOI: 10.1107/S2056989018010319/tx2007sup1.cif

Structure factors: contains datablock(s) I. DOI: 10.1107/S2056989018010319/tx2007Isup2.hkl

CCDC reference: 1856400

Additional supporting information:  crystallographic information; 3D view; checkCIF report

## Figures and Tables

**Table 1:** Selected geometric parameters (Å, °)

Cu1—O1	2.3570 (19)	Cu1—N3	2.0251 (18)
Cu1—N1	2.001 (2)	Cu1—N4	1.968 (2)
Cu1—N2	2.0311 (19)		
			
N1—Cu1—O1	81.40 (8)	N3—Cu1—O1	90.45 (7)
N1—Cu1—N2	84.06 (8)	N3—Cu1—N2	164.97 (8)
N1—Cu1—N3	80.92 (8)	N4—Cu1—O1	115.14 (8)
N2—Cu1—O1	87.91 (7)	N4—Cu1—N1	163.04 (9)

**Table 2:** Hydrogen-bond geometry and π–π stacking interactions (Å, °) *Cg*4, *Cg*5, *Cg*6 and *Cg*7 are the centroids of the N2/C4–C7/C12, N3/C14–C17/C22, C7–C12 and C17–C22 rings, respectively.

*D*—H⋯*A*	*D*—H	H⋯*A*	*D*⋯*A*	*D*—H⋯*A*
C1—H1*A*⋯O3^i^	0.97	2.68	3.402 (4)	132
C1—H1*B*⋯O9^ii^	0.97	2.57	3.397 (4)	143
C3—H3*B*⋯O8^iii^	0.97	2.58	3.446 (6)	148
C5—H5⋯O5^iv^	0.93	2.87	3.441 (6)	121
C6—H6⋯O5^iv^	0.93	2.70	3.366 (6)	129
C9—H9⋯O5*A* ^ii^	0.93	2.65	3.29 (4)	127
C10—H10⋯O2^ii^	0.93	2.77	3.427 (5)	128
C10—H10⋯O8*A* ^v^	0.93	2.64	3.329 (13)	131
C11—H11⋯N4	0.93	2.43	3.074 (3)	126
C11—H11⋯O8^v^	0.93	2.85	3.443 (6)	123
C13—H13*A*⋯O4	0.97	2.59	3.220 (3)	123
C13—H13*A*⋯O8^iii^	0.97	2.70	3.378 (7)	128
C15—H15⋯O2^i^	0.93	2.65	3.440 (5)	143
C15—H15⋯O2*A* ^i^	0.93	2.57	3.24 (3)	129
C18—H18⋯O4^v^	0.93	2.55	3.417 (3)	156
C20—H20⋯O6^v^	0.93	2.63	3.248 (8)	125
C21—H21⋯O6^v^	0.93	2.64	3.252 (8)	124
C23—H23*B*⋯O2^vi^	0.96	2.47	3.347 (6)	152
C8—H8⋯*Cg*7^vii^	0.93	2.75	3.511 (3)	139
C19—H19⋯*Cg*6^viii^	0.93	2.76	3.377 (3)	125
Cl1—O3⋯*Cg*4		3.43 (1)	4.2079 (13)	114 (1)
Cl1—O2*A*⋯*Cg*4		3.90 (5)	4.2079 (13)	89 (2)
*Cg*5⋯*Cg*5^v^			4.0264 (14)	
*Cg*5⋯*Cg*7^v^			3.7767 (14)	

Symmetry codes: (i) 
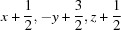
; (ii) 
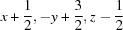
; (iii) 
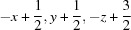
; (iv) 
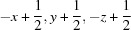
; (v) 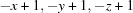; (vi) 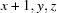; (vii) 
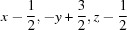
; (viii) 
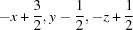
.

**Table 3:** Experimental details

Crystal data	
Chemical formula	[Cu(C_2_H_3_N)(C_23_H_23_N_3_O)](ClO_4_)_2_
*M*_r_	660.94
Crystal system, space group	Monoclinic, *P*2_1_/*n*
Temperature (K)	293
*a*, *b*, *c* (Å)	11.3597 (3), 16.9611 (4), 14.5514 (3)
β (°)	95.622 (2)
*V* (Å^3^)	2790.18 (11)
*Z*	4
Radiation type	Mo *K*α
μ (mm^−1^)	1.03
Crystal size (mm)	0.26 × 0.16 × 0.12

Data collection	
Diffractometer	Rigaku Oxford Diffraction Gemini Eos
Absorption correction	Multi-scan (*CrysAlis PRO*; Rigaku OD, 2015 ▸)
*T*_min_, *T*_max_	0.883, 1.000
No. of measured, independent and observed [*I* > 2σ(*I*)] reflections	21447, 9280, 6238
*R*_int_	0.028
(sin θ/λ)_max_ (Å^−1^)	0.762

Refinement	
*R*[*F* ^2^ > 2σ(*F* ^2^)], *wR*(*F* ^2^), *S*	0.050, 0.141, 1.02
No. of reflections	9280
No. of parameters	392
H-atom treatment	H-atom parameters constrained
Δρ_max_, Δρ_min_ (e Å^−3^)	0.63, −0.62

Computer programs: *CrysAlis PRO* (Rigaku OD, 2015 ▸), *SHELXT* (Sheldrick, 2015*b* ▸), *SHELXL* (Sheldrick, 2015*a* ▸) and *OLEX2* (Dolomanov *et al.*, 2009 ▸).

## supplementary crystallographic information

### Crystal data

**Table:**

[Cu(C_2_H_3_N)(C_23_H_23_N_3_O)](ClO_4_)_2_	*F*(000) = 1356
*M**_r_* = 660.94	*D*_x_ = 1.573 Mg m^−^^3^
Monoclinic, *P*2_1_/*n*	Mo *K*α radiation, λ = 0.71073 Å
*a* = 11.3597 (3) Å	Cell parameters from 4574 reflections
*b* = 16.9611 (4) Å	θ = 3.4–30.8°
*c* = 14.5514 (3) Å	µ = 1.03 mm^−^^1^
β = 95.622 (2)°	*T* = 293 K
*V* = 2790.18 (11) Å^3^	Prism, blue
*Z* = 4	0.26 × 0.16 × 0.12 mm

### Data collection

**Table:**

Rigaku Oxford Diffraction Gemini Eos diffractometer	9280 independent reflections
Radiation source: fine-focus sealed X-ray tube, Enhance (Mo) X-ray Source	6238 reflections with *I* > 2σ(*I*)
Graphite monochromator	*R*_int_ = 0.028
Detector resolution: 16.0416 pixels mm^-1^	θ_max_ = 32.8°, θ_min_ = 3.2°
ω scans	*h* = −17→17
Absorption correction: multi-scan (CrysAlisPro; Rigaku OD, 2015)	*k* = −25→16
*T*_min_ = 0.883, *T*_max_ = 1.000	*l* = −22→20
21447 measured reflections	

### Refinement

**Table:**

Refinement on *F*^2^	Primary atom site location: dual
Least-squares matrix: full	Hydrogen site location: inferred from neighbouring sites
*R*[*F*^2^ > 2σ(*F*^2^)] = 0.050	H-atom parameters constrained
*wR*(*F*^2^) = 0.141	*w* = 1/[σ^2^(*F*_o_^2^) + (0.0599*P*)^2^ + 1.6959*P*] where *P* = (*F*_o_^2^ + 2*F*_c_^2^)/3
*S* = 1.02	(Δ/σ)_max_ = 0.002
9280 reflections	Δρ_max_ = 0.63 e Å^−^^3^
392 parameters	Δρ_min_ = −0.62 e Å^−^^3^
0 restraints	

### Special details

**Table:**

Geometry. All esds (except the esd in the dihedral angle between two l.s. planes) are estimated using the full covariance matrix. The cell esds are taken into account individually in the estimation of esds in distances, angles and torsion angles; correlations between esds in cell parameters are only used when they are defined by crystal symmetry. An approximate (isotropic) treatment of cell esds is used for estimating esds involving l.s. planes.

### Fractional atomic coordinates and isotropic or equivalent isotropic displacement parameters (Å^2^)

**Table:**

	*x*	*y*	*z*	*U*_iso_*/*U*_eq_	Occ. (<1)
Cu1	0.55099 (2)	0.71839 (2)	0.33686 (2)	0.03253 (9)	
O1	0.75176 (16)	0.75392 (12)	0.33841 (14)	0.0470 (4)	
N1	0.55979 (18)	0.79512 (12)	0.44173 (14)	0.0367 (4)	
N2	0.51058 (16)	0.81527 (11)	0.25797 (13)	0.0336 (4)	
N3	0.59144 (16)	0.64254 (11)	0.44274 (13)	0.0322 (4)	
N4	0.49754 (19)	0.63548 (13)	0.24774 (14)	0.0415 (5)	
C1	0.6863 (2)	0.81542 (16)	0.4695 (2)	0.0463 (6)	
H1A	0.721959	0.773908	0.508802	0.056*	
H1B	0.689753	0.863821	0.505207	0.056*	
C2	0.7563 (2)	0.82575 (17)	0.3879 (2)	0.0507 (7)	
H2A	0.722827	0.867986	0.348680	0.061*	
H2B	0.837625	0.839065	0.408536	0.061*	
C3	0.4872 (3)	0.86476 (15)	0.41151 (19)	0.0466 (6)	
H3A	0.521876	0.911629	0.441284	0.056*	
H3B	0.408261	0.858502	0.430467	0.056*	
C4	0.4795 (2)	0.87510 (14)	0.30860 (18)	0.0389 (5)	
C5	0.4372 (2)	0.94704 (16)	0.2706 (2)	0.0483 (6)	
H5	0.419262	0.988444	0.308724	0.058*	
C6	0.4228 (2)	0.95522 (16)	0.1773 (2)	0.0488 (6)	
H6	0.392258	1.001824	0.151054	0.059*	
C7	0.4541 (2)	0.89326 (15)	0.12037 (19)	0.0397 (5)	
C8	0.4441 (2)	0.89886 (18)	0.0231 (2)	0.0497 (7)	
H8	0.411205	0.943900	−0.005389	0.060*	
C9	0.4815 (3)	0.83981 (18)	−0.0292 (2)	0.0512 (7)	
H9	0.472685	0.843926	−0.093188	0.061*	
C10	0.5335 (2)	0.77235 (17)	0.01305 (19)	0.0458 (6)	
H10	0.561192	0.732642	−0.023301	0.055*	
C11	0.5441 (2)	0.76417 (15)	0.10707 (18)	0.0390 (5)	
H11	0.579267	0.719209	0.134112	0.047*	
C12	0.50211 (19)	0.82345 (13)	0.16290 (16)	0.0332 (4)	
C13	0.5108 (2)	0.75237 (16)	0.51870 (17)	0.0426 (5)	
H13A	0.425154	0.751374	0.508990	0.051*	
H13B	0.533815	0.778812	0.576811	0.051*	
C14	0.5585 (2)	0.67007 (15)	0.52143 (16)	0.0366 (5)	
C15	0.5679 (2)	0.62524 (17)	0.60341 (17)	0.0451 (6)	
H15	0.545784	0.646621	0.658017	0.054*	
C16	0.6095 (2)	0.55061 (17)	0.60134 (18)	0.0461 (6)	
H16	0.612713	0.519495	0.654150	0.055*	
C17	0.6478 (2)	0.52007 (14)	0.52018 (17)	0.0385 (5)	
C18	0.6968 (2)	0.44325 (16)	0.5147 (2)	0.0486 (7)	
H18	0.699525	0.409785	0.565488	0.058*	
C19	0.7394 (3)	0.41860 (17)	0.4359 (2)	0.0539 (7)	
H19	0.769698	0.367859	0.432606	0.065*	
C20	0.7383 (2)	0.46862 (16)	0.3597 (2)	0.0478 (6)	
H20	0.770800	0.451518	0.306934	0.057*	
C21	0.6902 (2)	0.54223 (15)	0.36142 (17)	0.0386 (5)	
H21	0.689727	0.574828	0.309996	0.046*	
C22	0.64130 (19)	0.56881 (13)	0.44086 (16)	0.0332 (4)	
C23	0.8218 (3)	0.7556 (3)	0.2623 (2)	0.0666 (9)	
H23A	0.824998	0.703717	0.236289	0.100*	
H23B	0.900357	0.772868	0.283065	0.100*	
H23C	0.787033	0.791379	0.216179	0.100*	
C24	0.4563 (3)	0.58873 (19)	0.2001 (2)	0.0513 (7)	
C25	0.4030 (5)	0.5283 (3)	0.1392 (3)	0.1066 (17)	
H25A	0.403103	0.545212	0.076221	0.160*	
H25B	0.323120	0.519299	0.152765	0.160*	
H25C	0.447537	0.480328	0.148139	0.160*	
Cl1	0.21788 (6)	0.71417 (6)	0.27686 (5)	0.0607 (2)	
O2	0.1175 (4)	0.7570 (4)	0.2958 (3)	0.131 (2)	0.900 (10)
O2A	0.176 (4)	0.804 (4)	0.301 (3)	0.131 (2)	0.100 (10)
O3	0.2582 (2)	0.73903 (19)	0.19302 (15)	0.0759 (8)	
O4	0.31026 (19)	0.72130 (14)	0.35046 (14)	0.0593 (6)	
O5	0.1964 (8)	0.6280 (5)	0.2656 (5)	0.120 (3)	0.779 (16)
O5A	0.144 (3)	0.6625 (17)	0.2953 (18)	0.120 (3)	0.221 (16)
Cl2	0.23843 (8)	0.43937 (5)	0.92382 (6)	0.0615 (2)	
O6	0.2005 (11)	0.4593 (6)	0.8369 (4)	0.199 (4)	0.681 (7)
O6A	0.132 (2)	0.4040 (13)	0.8829 (9)	0.199 (4)	0.319 (7)
O7	0.3251 (8)	0.4841 (5)	0.8889 (7)	0.164 (3)	0.652 (7)
O7A	0.3697 (14)	0.4290 (10)	0.9530 (13)	0.164 (3)	0.348 (7)
O8	0.2279 (7)	0.3596 (3)	0.9373 (5)	0.130 (3)	0.700 (7)
O8A	0.3117 (19)	0.3946 (7)	0.9791 (12)	0.130 (3)	0.300 (7)
O9	0.1885 (3)	0.48505 (17)	0.9896 (2)	0.0946 (9)	

### Atomic displacement parameters (Å^2^)

**Table:**

	*U*^11^	*U*^22^	*U*^33^	*U*^12^	*U*^13^	*U*^23^
Cu1	0.04023 (16)	0.02837 (14)	0.02880 (14)	0.00429 (11)	0.00239 (10)	0.00066 (11)
O1	0.0397 (9)	0.0505 (10)	0.0513 (11)	0.0031 (8)	0.0075 (8)	0.0029 (9)
N1	0.0433 (11)	0.0329 (10)	0.0334 (10)	0.0046 (8)	0.0011 (8)	−0.0026 (8)
N2	0.0336 (9)	0.0309 (9)	0.0360 (10)	0.0039 (7)	0.0019 (7)	0.0020 (8)
N3	0.0352 (9)	0.0329 (9)	0.0279 (8)	−0.0001 (7)	0.0009 (7)	0.0015 (7)
N4	0.0485 (12)	0.0384 (10)	0.0363 (10)	0.0044 (9)	−0.0021 (9)	0.0012 (9)
C1	0.0501 (14)	0.0392 (13)	0.0479 (14)	−0.0032 (11)	−0.0042 (11)	−0.0070 (12)
C2	0.0440 (14)	0.0449 (14)	0.0618 (18)	−0.0057 (12)	−0.0021 (12)	0.0057 (13)
C3	0.0605 (16)	0.0357 (12)	0.0436 (14)	0.0153 (12)	0.0050 (12)	−0.0049 (11)
C4	0.0360 (11)	0.0347 (11)	0.0456 (13)	0.0077 (9)	0.0018 (10)	0.0000 (10)
C5	0.0489 (14)	0.0372 (12)	0.0583 (17)	0.0158 (11)	0.0036 (12)	−0.0011 (12)
C6	0.0442 (14)	0.0376 (13)	0.0631 (17)	0.0127 (11)	−0.0026 (12)	0.0099 (12)
C7	0.0285 (10)	0.0396 (12)	0.0497 (14)	0.0015 (9)	−0.0033 (9)	0.0095 (11)
C8	0.0444 (14)	0.0549 (16)	0.0470 (14)	0.0005 (12)	−0.0094 (11)	0.0176 (13)
C9	0.0503 (15)	0.0609 (17)	0.0404 (14)	−0.0093 (13)	−0.0053 (11)	0.0104 (13)
C10	0.0488 (14)	0.0499 (15)	0.0387 (13)	−0.0065 (12)	0.0046 (11)	−0.0004 (12)
C11	0.0426 (12)	0.0356 (11)	0.0388 (12)	−0.0006 (10)	0.0047 (10)	0.0040 (10)
C12	0.0281 (10)	0.0339 (11)	0.0373 (11)	−0.0016 (8)	0.0017 (8)	0.0034 (9)
C13	0.0505 (14)	0.0468 (13)	0.0310 (11)	0.0038 (12)	0.0063 (10)	−0.0066 (11)
C14	0.0361 (11)	0.0415 (12)	0.0317 (11)	−0.0038 (9)	0.0011 (9)	−0.0006 (10)
C15	0.0513 (14)	0.0561 (15)	0.0273 (11)	−0.0071 (12)	0.0015 (10)	0.0016 (11)
C16	0.0504 (14)	0.0526 (15)	0.0333 (12)	−0.0120 (12)	−0.0055 (10)	0.0145 (11)
C17	0.0362 (11)	0.0364 (11)	0.0404 (12)	−0.0066 (9)	−0.0090 (9)	0.0075 (10)
C18	0.0454 (14)	0.0374 (12)	0.0590 (17)	−0.0055 (11)	−0.0156 (12)	0.0149 (12)
C19	0.0480 (15)	0.0367 (13)	0.073 (2)	0.0064 (11)	−0.0151 (14)	0.0009 (14)
C20	0.0427 (13)	0.0437 (14)	0.0554 (16)	0.0095 (11)	−0.0040 (11)	−0.0063 (12)
C21	0.0363 (11)	0.0381 (12)	0.0403 (12)	0.0030 (10)	−0.0020 (9)	0.0007 (10)
C22	0.0313 (10)	0.0317 (10)	0.0352 (11)	−0.0021 (8)	−0.0038 (8)	0.0031 (9)
C23	0.0497 (17)	0.090 (2)	0.062 (2)	0.0048 (17)	0.0169 (14)	0.0101 (19)
C24	0.0553 (16)	0.0592 (17)	0.0383 (13)	−0.0059 (14)	−0.0014 (11)	−0.0038 (13)
C25	0.121 (4)	0.123 (4)	0.073 (3)	−0.044 (3)	0.001 (3)	−0.048 (3)
Cl1	0.0402 (3)	0.0946 (6)	0.0459 (4)	−0.0098 (4)	−0.0030 (3)	0.0243 (4)
O2	0.053 (2)	0.254 (6)	0.091 (2)	0.053 (3)	0.0304 (18)	0.067 (3)
O2A	0.053 (2)	0.254 (6)	0.091 (2)	0.053 (3)	0.0304 (18)	0.067 (3)
O3	0.0582 (13)	0.126 (2)	0.0432 (12)	0.0025 (14)	0.0045 (10)	0.0245 (14)
O4	0.0571 (12)	0.0754 (15)	0.0433 (11)	−0.0029 (10)	−0.0054 (9)	0.0094 (10)
O5	0.154 (6)	0.092 (4)	0.105 (4)	−0.068 (4)	−0.031 (4)	0.007 (3)
O5A	0.154 (6)	0.092 (4)	0.105 (4)	−0.068 (4)	−0.031 (4)	0.007 (3)
Cl2	0.0803 (5)	0.0500 (4)	0.0570 (4)	−0.0022 (4)	0.0211 (4)	−0.0034 (4)
O6	0.324 (12)	0.213 (8)	0.058 (3)	0.033 (8)	0.004 (4)	0.030 (4)
O6A	0.324 (12)	0.213 (8)	0.058 (3)	0.033 (8)	0.004 (4)	0.030 (4)
O7	0.162 (6)	0.138 (6)	0.213 (9)	−0.031 (5)	0.126 (7)	−0.003 (5)
O7A	0.162 (6)	0.138 (6)	0.213 (9)	−0.031 (5)	0.126 (7)	−0.003 (5)
O8	0.202 (7)	0.047 (2)	0.154 (5)	0.018 (3)	0.078 (5)	0.004 (3)
O8A	0.202 (7)	0.047 (2)	0.154 (5)	0.018 (3)	0.078 (5)	0.004 (3)
O9	0.109 (2)	0.0719 (17)	0.110 (2)	−0.0034 (16)	0.0437 (18)	−0.0223 (17)

### Geometric parameters (Å, º)

**Table:**

Cu1—O1	2.3570 (19)	C13—H13B	0.9700
Cu1—N1	2.001 (2)	C13—C14	1.496 (4)
Cu1—N2	2.0311 (19)	C14—C15	1.410 (3)
Cu1—N3	2.0251 (18)	C15—H15	0.9300
Cu1—N4	1.968 (2)	C15—C16	1.353 (4)
O1—C2	1.414 (4)	C16—H16	0.9300
O1—C23	1.426 (4)	C16—C17	1.397 (4)
N1—C1	1.494 (3)	C17—C18	1.422 (4)
N1—C3	1.482 (3)	C17—C22	1.416 (3)
N1—C13	1.487 (3)	C18—H18	0.9300
N2—C4	1.322 (3)	C18—C19	1.353 (5)
N2—C12	1.384 (3)	C19—H19	0.9300
N3—C14	1.324 (3)	C19—C20	1.396 (4)
N3—C22	1.374 (3)	C20—H20	0.9300
N4—C24	1.124 (3)	C20—C21	1.364 (4)
C1—H1A	0.9700	C21—H21	0.9300
C1—H1B	0.9700	C21—C22	1.405 (3)
C1—C2	1.503 (4)	C23—H23A	0.9600
C2—H2A	0.9700	C23—H23B	0.9600
C2—H2B	0.9700	C23—H23C	0.9600
C3—H3A	0.9700	C24—C25	1.449 (5)
C3—H3B	0.9700	C25—H25A	0.9600
C3—C4	1.502 (4)	C25—H25B	0.9600
C4—C5	1.405 (3)	C25—H25C	0.9600
C5—H5	0.9300	Cl1—O2	1.402 (4)
C5—C6	1.359 (4)	Cl1—O2A	1.65 (6)
C6—H6	0.9300	Cl1—O3	1.409 (2)
C6—C7	1.405 (4)	Cl1—O4	1.429 (2)
C7—C8	1.412 (4)	Cl1—O5	1.489 (8)
C7—C12	1.420 (3)	Cl1—O5A	1.26 (2)
C8—H8	0.9300	Cl2—O6	1.339 (6)
C8—C9	1.351 (4)	Cl2—O6A	1.43 (2)
C9—H9	0.9300	Cl2—O7	1.379 (6)
C9—C10	1.402 (4)	Cl2—O7A	1.520 (18)
C10—H10	0.9300	Cl2—O8	1.375 (5)
C10—C11	1.369 (4)	Cl2—O8A	1.336 (17)
C11—H11	0.9300	Cl2—O9	1.394 (3)
C11—C12	1.405 (3)	O6—O7	1.595 (12)
C13—H13A	0.9700	O7A—O8A	0.984 (17)
			
N1—Cu1—O1	81.40 (8)	N1—C13—H13B	110.0
N1—Cu1—N2	84.06 (8)	N1—C13—C14	108.3 (2)
N1—Cu1—N3	80.92 (8)	H13A—C13—H13B	108.4
N2—Cu1—O1	87.91 (7)	C14—C13—H13A	110.0
N3—Cu1—O1	90.45 (7)	C14—C13—H13B	110.0
N3—Cu1—N2	164.97 (8)	N3—C14—C13	116.0 (2)
N4—Cu1—O1	115.14 (8)	N3—C14—C15	122.5 (2)
N4—Cu1—N1	163.04 (9)	C15—C14—C13	121.5 (2)
N4—Cu1—N2	99.65 (8)	C14—C15—H15	120.5
N4—Cu1—N3	94.57 (8)	C16—C15—C14	118.9 (2)
C2—O1—Cu1	102.24 (15)	C16—C15—H15	120.5
C2—O1—C23	112.5 (3)	C15—C16—H16	119.9
C23—O1—Cu1	127.62 (19)	C15—C16—C17	120.3 (2)
C1—N1—Cu1	109.34 (15)	C17—C16—H16	119.9
C3—N1—Cu1	107.90 (15)	C16—C17—C18	122.9 (2)
C3—N1—C1	112.9 (2)	C16—C17—C22	118.5 (2)
C3—N1—C13	112.0 (2)	C22—C17—C18	118.6 (3)
C13—N1—Cu1	105.24 (15)	C17—C18—H18	119.8
C13—N1—C1	109.2 (2)	C19—C18—C17	120.3 (3)
C4—N2—Cu1	111.34 (16)	C19—C18—H18	119.8
C4—N2—C12	118.9 (2)	C18—C19—H19	119.7
C12—N2—Cu1	129.47 (16)	C18—C19—C20	120.6 (3)
C14—N3—Cu1	111.82 (16)	C20—C19—H19	119.7
C14—N3—C22	119.3 (2)	C19—C20—H20	119.5
C22—N3—Cu1	128.77 (15)	C21—C20—C19	120.9 (3)
C24—N4—Cu1	173.1 (2)	C21—C20—H20	119.5
N1—C1—H1A	109.1	C20—C21—H21	120.0
N1—C1—H1B	109.1	C20—C21—C22	120.0 (2)
N1—C1—C2	112.5 (2)	C22—C21—H21	120.0
H1A—C1—H1B	107.8	N3—C22—C17	120.2 (2)
C2—C1—H1A	109.1	N3—C22—C21	120.4 (2)
C2—C1—H1B	109.1	C21—C22—C17	119.4 (2)
O1—C2—C1	107.9 (2)	O1—C23—H23A	109.5
O1—C2—H2A	110.1	O1—C23—H23B	109.5
O1—C2—H2B	110.1	O1—C23—H23C	109.5
C1—C2—H2A	110.1	H23A—C23—H23B	109.5
C1—C2—H2B	110.1	H23A—C23—H23C	109.5
H2A—C2—H2B	108.4	H23B—C23—H23C	109.5
N1—C3—H3A	109.3	N4—C24—C25	179.7 (4)
N1—C3—H3B	109.3	C24—C25—H25A	109.5
N1—C3—C4	111.4 (2)	C24—C25—H25B	109.5
H3A—C3—H3B	108.0	C24—C25—H25C	109.5
C4—C3—H3A	109.3	H25A—C25—H25B	109.5
C4—C3—H3B	109.3	H25A—C25—H25C	109.5
N2—C4—C3	118.3 (2)	H25B—C25—H25C	109.5
N2—C4—C5	123.2 (2)	O2—Cl1—O3	110.8 (2)
C5—C4—C3	118.6 (2)	O2—Cl1—O4	111.1 (2)
C4—C5—H5	120.5	O2—Cl1—O5	113.7 (5)
C6—C5—C4	119.0 (3)	O3—Cl1—O2A	91.7 (14)
C6—C5—H5	120.5	O3—Cl1—O4	110.24 (14)
C5—C6—H6	120.0	O3—Cl1—O5	105.2 (4)
C5—C6—C7	120.0 (2)	O4—Cl1—O2A	88.1 (16)
C7—C6—H6	120.0	O4—Cl1—O5	105.4 (3)
C6—C7—C8	122.7 (2)	O5A—Cl1—O2A	113 (3)
C6—C7—C12	118.4 (2)	O5A—Cl1—O3	132.2 (12)
C8—C7—C12	118.9 (2)	O5A—Cl1—O4	110.9 (11)
C7—C8—H8	119.5	O6—Cl2—O7	71.9 (6)
C9—C8—C7	121.0 (3)	O6—Cl2—O8	111.1 (6)
C9—C8—H8	119.5	O6—Cl2—O9	113.1 (4)
C8—C9—H9	120.0	O6A—Cl2—O7A	146.7 (10)
C8—C9—C10	120.0 (3)	O7—Cl2—O9	107.5 (4)
C10—C9—H9	120.0	O8—Cl2—O7	132.0 (4)
C9—C10—H10	119.6	O8—Cl2—O9	113.7 (3)
C11—C10—C9	120.9 (3)	O8A—Cl2—O6A	117.7 (11)
C11—C10—H10	119.6	O8A—Cl2—O7A	39.6 (8)
C10—C11—H11	119.9	O8A—Cl2—O9	100.0 (6)
C10—C11—C12	120.2 (2)	O9—Cl2—O6A	97.7 (8)
C12—C11—H11	119.9	O9—Cl2—O7A	109.1 (6)
N2—C12—C7	120.4 (2)	Cl2—O6—O7	55.2 (4)
N2—C12—C11	120.8 (2)	Cl2—O7—O6	52.9 (4)
C11—C12—C7	118.8 (2)	O8A—O7A—Cl2	60.1 (15)
N1—C13—H13A	110.0	O7A—O8A—Cl2	80.3 (16)
			
Cu1—O1—C2—C1	43.6 (2)	C9—C10—C11—C12	0.4 (4)
Cu1—N1—C1—C2	40.5 (3)	C10—C11—C12—N2	178.9 (2)
Cu1—N1—C3—C4	−26.8 (3)	C10—C11—C12—C7	−3.1 (3)
Cu1—N1—C13—C14	42.2 (2)	C12—N2—C4—C3	179.0 (2)
Cu1—N2—C4—C3	4.3 (3)	C12—N2—C4—C5	0.5 (4)
Cu1—N2—C4—C5	−174.2 (2)	C12—C7—C8—C9	−1.3 (4)
Cu1—N2—C12—C7	169.80 (16)	C13—N1—C1—C2	155.2 (2)
Cu1—N2—C12—C11	−12.2 (3)	C13—N1—C3—C4	−142.2 (2)
Cu1—N3—C14—C13	−5.9 (3)	C13—C14—C15—C16	178.8 (2)
Cu1—N3—C14—C15	174.49 (19)	C14—N3—C22—C17	5.5 (3)
Cu1—N3—C22—C17	−171.17 (16)	C14—N3—C22—C21	−172.1 (2)
Cu1—N3—C22—C21	11.2 (3)	C14—C15—C16—C17	3.0 (4)
N1—C1—C2—O1	−60.0 (3)	C15—C16—C17—C18	177.4 (2)
N1—C3—C4—N2	15.5 (3)	C15—C16—C17—C22	−0.3 (4)
N1—C3—C4—C5	−166.0 (2)	C16—C17—C18—C19	−175.7 (2)
N1—C13—C14—N3	−24.7 (3)	C16—C17—C22—N3	−4.0 (3)
N1—C13—C14—C15	154.9 (2)	C16—C17—C22—C21	173.6 (2)
N2—C4—C5—C6	2.6 (4)	C17—C18—C19—C20	1.4 (4)
N3—C14—C15—C16	−1.6 (4)	C18—C17—C22—N3	178.1 (2)
C1—N1—C3—C4	94.1 (3)	C18—C17—C22—C21	−4.2 (3)
C1—N1—C13—C14	−75.1 (2)	C18—C19—C20—C21	−2.6 (4)
C3—N1—C1—C2	−79.6 (3)	C19—C20—C21—C22	0.3 (4)
C3—N1—C13—C14	159.2 (2)	C20—C21—C22—N3	−179.2 (2)
C3—C4—C5—C6	−175.9 (3)	C20—C21—C22—C17	3.1 (3)
C4—N2—C12—C7	−3.8 (3)	C22—N3—C14—C13	176.9 (2)
C4—N2—C12—C11	174.2 (2)	C22—N3—C14—C15	−2.7 (3)
C4—C5—C6—C7	−2.2 (4)	C22—C17—C18—C19	2.0 (4)
C5—C6—C7—C8	−178.4 (3)	C23—O1—C2—C1	−176.4 (2)
C5—C6—C7—C12	−0.9 (4)	O6A—Cl2—O7A—O8A	−59 (2)
C6—C7—C8—C9	176.1 (3)	O6A—Cl2—O8A—O7A	147.8 (14)
C6—C7—C12—N2	4.0 (3)	O8—Cl2—O6—O7	−129.0 (5)
C6—C7—C12—C11	−174.0 (2)	O8—Cl2—O7—O6	102.4 (7)
C7—C8—C9—C10	−1.4 (4)	O9—Cl2—O6—O7	101.7 (5)
C8—C7—C12—N2	−178.4 (2)	O9—Cl2—O7—O6	−109.3 (5)
C8—C7—C12—C11	3.5 (3)	O9—Cl2—O7A—O8A	82.6 (13)
C8—C9—C10—C11	1.9 (4)	O9—Cl2—O8A—O7A	−107.9 (12)

### Hydrogen-bond geometry (Å, º)

Cg4, Cg5, Cg6 and Cg7 are the centroids of the N2/C4–C7/C12, N3/C14–C17/C22, C7–C12 and C17–C22 rings, respectively.

**Table:**

*D*—H···*A*	*D*—H	H···*A*	*D*···*A*	*D*—H···*A*
C1—H1*A*···O3^i^	0.97	2.68	3.402 (4)	132
C1—H1*B*···O9^ii^	0.97	2.57	3.397 (4)	143
C3—H3*B*···O8^iii^	0.97	2.58	3.446 (6)	148
C5—H5···O5^iv^	0.93	2.87	3.441 (6)	121
C6—H6···O5^iv^	0.93	2.70	3.366 (6)	129
C9—H9···O5*A*^ii^	0.93	2.65	3.29 (4)	127
C10—H10···O2^ii^	0.93	2.77	3.427 (5)	128
C10—H10···O8*A*^v^	0.93	2.64	3.329 (13)	131
C11—H11···N4	0.93	2.43	3.074 (3)	126
C11—H11···O8^v^	0.93	2.85	3.443 (6)	123
C13—H13*A*···O4	0.97	2.59	3.220 (3)	123
C13—H13*A*···O8^iii^	0.97	2.70	3.378 (7)	128
C15—H15···O2^i^	0.93	2.65	3.440 (5)	143
C15—H15···O2*A*^i^	0.93	2.57	3.24 (3)	129
C18—H18···O4^v^	0.93	2.55	3.417 (3)	156
C20—H20···O6^v^	0.93	2.63	3.248 (8)	125
C21—H21···O6^v^	0.93	2.64	3.252 (8)	124
C23—H23*B*···O2^vi^	0.96	2.47	3.347 (6)	152
C8—H8···*Cg*7^vii^	0.93	2.75	3.511 (3)	139
C19—H19···*Cg*6^viii^	0.93	2.76	3.377 (3)	125
Cl1—O3···*Cg*4		3.43 (1)	4.2079 (13)	114 (1)
Cl1—O2*A*···*Cg*4		3.90 (5)	4.2079 (13)	89 (2)
*Cg*5···*Cg*5^v^			4.0264 (14)	
*Cg*5···*Cg*7^v^			3.7767 (14)	

Symmetry codes: (i) *x*+1/2, −*y*+3/2, *z*+1/2; (ii) *x*+1/2, −*y*+3/2, *z*−1/2; (iii) −*x*+1/2, *y*+1/2, −*z*+3/2; (iv) −*x*+1/2, *y*+1/2, −*z*+1/2; (v) −*x*+1, −*y*+1, −*z*+1; (vi) *x*+1, *y*, *z*; (vii) *x*−1/2, −*y*+3/2, *z*−1/2; (viii) −*x*+3/2, *y*−1/2, −*z*+1/2.
